# Effect of Precuring Warming on Mechanical Properties of Restorative Composites

**DOI:** 10.1155/2011/536212

**Published:** 2011-10-27

**Authors:** Kareem Nada, Omar El-Mowafy

**Affiliations:** Department of Clinical Sciences, Faculty of Dentistry, University of Toronto, 124 Edward Street, Toronto, ON, Canada M5G 1G6

## Abstract

To investigate the effect of prepolymerization warming on composites' mechanical properties, three composites were evaluated: Clearfil Majesty (CM) (Kuraray), Z-100 (3M/ESPE), and Light-Core (LC) (Bisco). Specimens were prepared from each composite at room temperature as control and 2 higher temperatures (37°C and 54°C) to test surface hardness (SH), compressive strength (CS), and diametral tensile strength (DTS). Data were statistically analyzed using ANOVA and Fisher's LSD tests. Results revealed that prewarming CM and Z100 specimens significantly improved their SH mean values (*P* < 0.05). Prewarming also improved mean CS values of Z100 specimens (*P* < 0.05). Furthermore, DTS mean value of CM prepared at 52° was significantly higher than that of room temperature specimens (*P* < 0.05). KHN, CS, and DTS mean values varied significantly among the three composites. In conclusion, Prewarming significantly enhanced surface hardness of 2 composites. Prewarming also improved bulk properties of the composites; however, this improvement was significant in only some of the tested materials.

## 1. Introduction

The use of resin composite as a substitute for amalgam in posterior restorations is on the rise [1]. This is due to an increase in aesthetic demands of patients and environmental concerns about mercury in amalgam. In addition, improvements in composites mechanical properties and reduced polymerization shrinkage that were achieved over the last decade by manufacturers encouraged clinicians to use composite more frequently in posterior restorations. Manufacturers' improvements were aimed at the microstructure of the material including monomer composition, size, shape, and distribution of inorganic filler particles and mainly targeted the filler loading percentage. Increasing the filler percentage resulted in higher viscosity [2] which added to the inherently viscous and sticky nature of some composites. As a result, concerns about handling, packing, and adaptation were aroused. Many attempts were made to enhance adaptation and decrease microleakage either by incorporating flowable composite [3–7], fiber inserts [4, 7, 8], or chemical and laser treatments of dentin [9].

Chairside warming of composite resins before photopolymerization is one of the recent trends in their application. Preheating reduces viscosity and increases flowability, which facilitates better adaptation to cavity walls [10, 11]. This reduces microleakage and thus the durability of the restoration [12, 13] and results in superior marginal adaptation [14, 15]. The increase in temperature of composite enhances both radical and monomer mobility, resulting in high degree of monomer conversion [16, 17] as well as improvement of polymerization rate [18]. As a result, more highly crosslinked polymer networking and improved mechanical and physical properties may be anticipated [19]. Superior surface hardness and greater depth of cure are also positive outcomes of preheating [10, 20].

Therefore, studying mechanical properties of the preheated composites is essential to understand the effect of heat on material's ability to withstand forces of mastication and to resist fracture and wear. Clinical data indicate that bulk fracture is one of two major challenges of composite restorations (the second being secondary caries) [18]. 

Available data about the effect of preheating on mechanical properties of composites are scarce and perhaps nonconclusive as Deb et al. reported that some of evaluated composites showed significantly higher flexural strength with preheating [11]. On the other hand, two other studies found no significant difference in flexural strength between heated and nonheated composites [14, 21]. It is worth mentioning that those three studies evaluated different composite brands and this may explain different findings as different brands may behave differently due to differences in monomer composition and fillers type and size. As yet, it seems that no studies were conducted to evaluate the effect of preheating on compressive and tensile strength of resin composites. Issues related to the use of composite preheating need to be investigated so that the clinician can better understand the variables associated with this method [22].

 The aim of the current study was to investigate the effect of precuring warming of composites at two different temperatures (37°C and 54°C) on their surface hardness (SH), compressive strength (CS), and diametral tensile strength (DTS). It was hypothesized that increasing the precuring temperature would increase both surface and bulk properties of the tested resins. 

## 2. Materials and Methods

Three restorative composites were evaluated in this investigation and their details are given in Table 1. Specimens were fabricated for three different mechanical tests. For each test one group of specimens was prepared at room temperature (21°C ± 1) as control and two groups were prepared after composites were pre-warmed to 37°C and 54°C. 

### 2.1. Microhardness Test 

**Figure 1:**
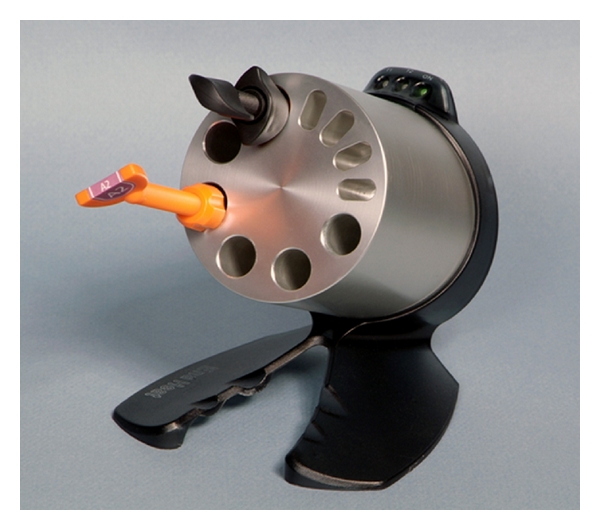
Composite warmer (Micerium S.p.A).

Twelve specimens (2 mm thick and 4 mm in diameter) were prepared from each composite at room temperature (RT), 37°C (T1) and 52°C (T2) forming 3 subgroups. Room temperature was standardized through the central climate control unit of the building. Composites were pre-warmed using composite heating conditioner (Ena Heat, Micerium S.p.A., Avegno GE, Italy) (Figure 1). Specimens were then light-polymerized for 40 s from upper surface only using LED unit with light intensity of 1100 mW/cm^2^ (Demi Plus, Kerr Corporation, Orange CA, USA). Using a microhardness tester with a Knoop indenter (Tukon 300, Acco Industries Inc., Wilson instruments division, Bridgeport CT, USA), 4 Knoop Hardness Number (KHN) readings were recorded for each specimen (*n* = 16) under 100 g load.

### 2.2. Compressive Strength Test

Using split Teflon moulds, 30 cylindrical specimens (6 mm high and 3 mm in diameter) were prepared from each composite at RT, T1, and T2 making 3 subgroups (*n* = 10) as above. Specimens were light-polymerized for 40 s from both ends then loaded in Instron machine (Universal Testing Machine, Instron Corporation, Canton, MA, USA) at 0.5 mm/min until failure. CS values were then calculated.

### 2.3. Diametral Tensile Strength Test

Thirty cylindrical specimens (3 mm thick and 3 mm in diameter) were prepared from each composite at RT, T1, and T2 forming 3 subgroups (*n* = 10) as above using the same method as compressive strength. Specimens were light-polymerized for 40 s from both ends then loaded sideways in Instron machine at 0.5 mm/min until failure. DTS values were then calculated. 

Filled molds of all specimens were compressed between 2 glass slides lined with transparent plastic sheet to achieve specimens with a uniform and smooth surface finish. For all three tests, specimens were stored in incubator at 37°C for 24 h before mechanical testing. 

Data were statistically analyzed with ANOVA and Fisher's LSD tests using SPSS software (SPSS Statistics 17.0, SPSS Inc., Chicago, IL, USA).

## 3. Results 

Table 2 shows mean KHN values for each material under each condition. ANOVA revealed significant differences among mean values of three materials. Further, post hoc Fisher's LSD test showed that preheated CM and Z100 specimens had mean hardness values that were significantly higher than that of RT (*P* < 0.05). 

Mean and standard deviation values for compressive strengths of tested composites are shown in Table 3. ANOVA revealed significant differences in mean CS values among 3 materials. Further post hoc Fisher's LSD test showed that T1 and T2 for Z100 had mean values that were significantly higher than that of RT (*P* = 0.028 and *P* = 0.019, resp.).

Mean and standard deviation values for diametral tensile strengths of tested composites are shown in Table 4. ANOVA revealed significant differences among means of three materials. Further, post hoc Fisher's LSD test showed that mean of T2 for CM was significantly higher than that of RT (*P* = 0.018). For LC and Z100, there were no significant differences in the means in spite of increased mean values with higher temperatures (*P* > 0.05).

## 4. Discussion

Three composite materials were evaluated; 2 are restorative composites (CM, Z100) and the other is core build-up material (LC). The choice of LC was based on the fact that composites are widely used as a core material. In Addition, studying the effect of prewarming on mechanical properties will provide useful information to practitioners who consider using this technique to increase flowability of composite core materials. The present study showed statistically significant differences in the favor of preheated specimens of CM and Z100 for surface hardness. The enhancement in the surface hardness most probably is due to a higher rate of conversion (caused by higher temperature) that resulted in highly crosslinked network [10, 20]. Polymerization rate and overall monomer conversion were reported to be higher at the top surface of preheated specimen [19]. This further explains the superior hardness with pre-warming. However, this was not the case with LC specimens as there were no significant differences between preheated and room temperature specimens. This may be explained by the chemical composition of the composite especially the monomer nature. As shown in Table 1, LC is the only tested material that does not contain TEGDMA in its BisGMA-based monomer. TEGDMA is widely used in dental composites to lower the viscosity of the inherently viscous BISGMA. Increasing content of TEGDMA in the monomer was reported to increase degree of conversion of the resin composites [23–25]. Instead of TEGDMA, LC has Ethoxylated BisGMA which is believed to have high polymerization rate. But it seems that temperature has an effect on TEGDMA and not on Ethoxylated BisGMA. LC has lower filler loading percentage than CM and Z100. Lower filler loading may not provide enough support to highly crosslinked network formed with the help of preheating. In other words, it is not enough for surface hardness improvement to have higher crosslinked network without high filler support. It was reported that filler mass fraction plays a role in composite's surface and mechanical properties [26, 27]. There were no significant differences in SH means between specimens heated to 37°C and 52°C. This reflects that warming temperature is not critical to achieve superior SH for CM and Z100. This finding is in agreement with Muñoz et al. who reported increase in surface hardness with preheating to different temperatures 37 and 60°C; however, different composites were tested [20].

Z100 was the only material that had significantly higher compressive strength with preheating as compared to ambient temperature. While CM and LC showed no significance in the CS mean values. This may be due to different filler content that caused these two materials to behave differently when prewarmed. The cooling effect that happens after removing the composite from the warming device might be another reason. In addition, compatbility of each material also plays a major role in the compressive strength which may further explain this finding. 

DTS of all materials showed increase in values with elevated temperature. However; this increase was only significant in the case of CM-T2. The wide scatter of data, however, might be the reason behind this statistical insignificance of the other groups. Similar observation was reported in two previous studies where authors obtained high standard deviations for flexural strength of composites [11, 28]. One of these studies investigated the effect of pre-warming on flexural strength and found significance in some but not all tested composites [11]. In contrast Fróes-Salgado et al. and Uctasli et al. reported no significant difference in flexural strength between preheated and room temperature composite [14, 21]. Further investigation is warranted in this respect. 

The positive outcomes of composite pre-warming, in the clinical situation, are expected to depend on the rate of cooling after removing from the warming device and on the handling time before curing. A previous study reported a 50% temperature drop within 2 min of removing composite compules from all of three heating devices tested [22]. Positive outcomes also depend on the composite brand and type as the current study showed. It is also worth mentioning that although all 3 tested materials showed different responses to heat treatment, none of these responses was negative. Either statistically significant improvement to mechanical properties or no statistical significance was found. 

In addition to the potential benefits of composite pre-warming, Daronch et al. reported that preheated composite allows for reduced light exposure time up to 75%, resulting in similar or better monomer conversion when compared to room temperature composite with regular exposure time [16]. This may reduce temperature generated from application of curing light. Intrapulpal temperature rise generated from using pre-warmed composite in vital teeth should not be a concern for clinicians. As a previous study reported, temperature rise was mild, while the greatest temperature change occurred with application of the curing light. When composite was preheated to 54°C or 60°C and placed on 1 mm thickness of remaining dentin, the temperature rise inside pulp was 0.8°C while the rise due to light curing was 5°C [29].

## 5. Conclusions

Within the limits of this study, the following can be concluded.

1. Prepolymerization warming of composites significantly enhanced surface hardness of two of the three tested composites. This may influence their wear-resistance. However, more investigation is needed in this respect.
2. Prepolymerization warming of composites significantly enhanced compressive strength of one tested material. Diametral tensile strength was increased with prepolymerization warming; however, the difference was statistically significant with only one material.
3. Different composite brands behave differently with heat treatment resulting in different mechanical properties.

## Figures and Tables

**Table 1:** Composite brands tested in the study and their monomer composition according to manufacturers' data.

Material	Clearfil Majesty	Z100	Light Core
Code	CM	Z100	LC
Filler size	0.02–1.5 *μ*m	0.01–3.5 *μ*m	0.04–9 *μ*m
Filler loading	92 wt%–82 vol%	85 wt%—66 vol%	80.5 wt%—65 vol%
Monomer Composition	BisGMA <3%: TEGDMA <3%	BisGMA 1–10%: TEGDMA 1–10%	BisGMA >5%: Ethoxylated BisGMA >1%
Manufacturer	Kuraray	3M/ESPE	Bisco
Shade	A2	A2	Blue
Lot Number	018AA	N157247	1000005651

**Table 2:** Means and SDs values of KHN for the three composite materials. Means with same superscript letter did not significantly differ statistically (*P* > 0.05).

	RT	T1	T2
CM	111.49^A^ (5.28)	118.50^B^ ( 8.92)	116.65^B^ (3.90)
LC	70.41^C^ (4.53)	69.05^C^ (2.53)	68.93^C^ (2.93)
Z100	96.31^D^ (3.00)	100.04^E^ (3.28)	101.71^E^ (1.80)

**Table 3:** Means and SDs of Compressive strength values for the three composite materials in MPa. Means with same superscript letter did not significantly differ statistically (*P* > 0.05).

	RT	T1	T2
CM	341.30^A^ (55.71)	306.64^A^ (26.91)	369.75^A^ (36.86)
LC	212.98**^B^** (26.22)	189.24^B^ (24.39)	204.14^B^ (20.25)
Z100	318.71^C^ (37.15)	351.2^D^ (30.03)	353.39^D^ (25.99)

**Table 4:** Means and SDs of diametral tensile strength values for the three composite materials in MPa. Means with same superscript letter did not significantly differ statistically (*P* > 0.05).

	RT	T1	T2
CM	53.75^A^ (16.83)	58.63^AB^ (12.39)	65.73^B^ (16.74)
LC	36.17^C^ (6.61)	43.46^C^ (3.52)	43.19^C^ (6.75)
Z100	57.75^D^ (5.61)	60.54^D^ (12.22)	63.97^D^ (8.93)
